# Label-free imaging flow cytometry for cell classification based directly on multiple off-axis holographic projections

**DOI:** 10.1117/1.JBO.30.1.016007

**Published:** 2025-01-23

**Authors:** Dana Aharoni, Matan Dudaie, Itay Barnea, Natan Tzvi Shaked

**Affiliations:** Tel Aviv University, Department of Biomedical Engineering, Faculty of Engineering, Tel Aviv, Israel

**Keywords:** digital holography, imaging flow cytometry, deep learning

## Abstract

**Significance:**

Imaging flow cytometry allows highly informative multi-point cell analysis for biological assays and medical diagnosis. Rapid processing of the imaged cells during flow allows real-time classification and sorting of the cells. Off-axis holography enables imaging flow cytometry without chemical cell staining but requires digital processing to the optical path delay profile for each frame before the cells can be classified, which slows down the overall processing throughput. We present a method for real-time cell classification via label-free quantitative imaging flow cytometry using digital holography, offering a comprehensive representation of cellular structures, without the need for digital processing before automatic cell classification.

**Aim:**

We aim to develop an automatic cell classification scheme based directly on the off-axis holographic projections of the cells during flow and test it for stain-free imaging flow cytometry of white blood cells.

**Approach:**

After building a dedicated off-axis holographic microscopy system for acquiring white blood cells during flow, we apply deep-learning classification directly in the off-axis hologram space, rather than in the quantitative phase profile space. This way, we simplify computational processes and allow a significant increase in the cell classification throughput. In addition, by utilizing multiple-viewpoint holographic projections of the cells rotated during flow, instead of using a single projection, we obtain better classification results due to the additional cellular information gained.

**Results:**

Our technique demonstrates increasing accuracy with additional viewpoint holographic projections from the optical system, achieving a 7.69% improvement when processing ten interferometric projections compared with a single interferometric projection (regular off-axis hologram). Our technique also outperforms using multiple optical path delay profile projections, requiring off-axis holographic digital preprocessing, by 17.95%, because the holographic projections are analyzed directly without preprocessing and includes the amplitude information as well.

**Conclusions:**

Our cell classification approach has great potential for high-throughput, high-content, label-free imaging flow cytometry for classification of large-scale cellular datasets and real-time cell classification during flow in clinical settings.

## 1 Introduction

Characterization and classification of biological cells are essential in diverse biomedical fields such as pathology, immunology, and diagnosis of various diseases. Flow cytometry is a technique used to identify and analyze individual cells by typically illuminating them with one or more light sources as they flow in a liquid stream. As each cell passes through the light beam, the system measures the light that the cells scatter in different directions: forward scatter, which indicates cell relative size, and side scatter, which provides insights into cell structural complexity. Fluorescent labeling is largely used in flow cytometry to improve the molecular specificity of the measured cells.^1^^,^^2^ Imaging flow cytometry (IFC) enhances traditional flow cytometry by combining multipoint imaging capabilities providing high-throughput cell analysis with spatial information analysis.^3^ The combination of IFC with fluorescence provides physical and chemical information of the imaged cells. However, fluorescent labeling may alter the cell viability or compromise the integrity of the cell, making it unsuitable for further processing of the cell in its environment. In addition, fluorescent labeling is not available to all cell types and is not suitable for cells whose surface antigens tend to change frequently.^4^^,^^5^

Digital holography enables cell imaging without the need for chemical labeling by capturing both amplitude and phase information. It produces quantitative phase images containing measurements of cell thickness and refractive index, providing insights into morphology and dynamics.^6^^,^^7^ Off-axis digital holography is based on the interference between a beam passing through a sample and a reference beam. The reconstructed quantitative phase profile of the cell is proportional to the optical phase delay (OPD) profile of the cell. Per each spatial (x,y) point, the OPD is defined as the product of the cell thickness and the difference between the integral refractive indices of the cell and its surrounding medium.^8^^–^^10^ In off-axis holography, where there is an angle between the sample and the reference beams, a digital Fourier-transform analysis with spatial filtering is typically performed to retrieve the OPD profile, followed by a phase unwrapping algorithm to solve 2π ambiguities,^11^ which is a time-consuming process that can be performed in tens of frames per second,^12^^,^^13^ rather than thousands of frames per second as required in imaging flow cytometry. The OPD profile contains the integral (axially averaged) refractive index of the cell, rather than its full three-dimensional (3D) refractive index distribution tomographic phase microscopy (TPM) overcomes this limitation by quantitatively measuring the 3D distribution of cell refractive indices. This is achieved by imaging the cell from different illumination angles using interferometry, followed by extensive digital processing of all holographic projections to the 3D refractive index map of the cell. The illumination angle can be obtained by rotating the sample with a fixed illumination beam or by fixing the sample and rotating the illumination beam.^14^^,^^15^ The lack of full angular coverage and inaccuracy in the projection angle for each image may result in compromised 3D reconstruction accuracy.^16^^,^^17^ The reconstruction process in TPM is computationally heavy and therefore unsuitable for handling large cell datasets or for real-time 3D cell visualization.^18^

In imaging flow cytometry, machine learning and deep learning models have been proven to be powerful tools for cell identification and classification tasks.^19^^–^^25^ These models have been investigated in conjunction with digital holography^26^^,^^27^ and TPM.^28^^,^^29^

In this paper, we propose a new method to classify biological cells based directly on their holographic projections acquired during rapid cell flow, without processing to the OPD profile. Instead of using the OPD profile per cell, which lacks amplitude information, we directly utilize the raw holograms themselves that contain both the amplitude and OPD profile in a single image.^30^ Furthermore, by employing multiple projections of the cells, obtained by randomly rolling the cells during flow,^31^ but without reconstructing the 3D refractive-index map as done in TPM, we significantly reduce computational complexity. Our work is the first to suggest automatic cell classification both (1) in the raw-hologram domain and (2) on multiple projections together, simple to obtain conditions in imaging flow cytometry. We demonstrate superior model accuracy while (1) simplifying computational processes by minimized preprocessing steps and (2) enabling highly informative label-free imaging flow cytometry by processing multiple viewing perspectives of the cell while flowing, paving the way for potential real-time cell classification in imaging flow cytometry. Using a convolutional neural network for cell classification in the holographic projections space, we classify three types of T-cells, demonstrating that the accuracy increases as additional holographic projections are used, as well as superiority when processing the holographic projections rather than the OPD projections.

## 2 Sample Preparation

Blood was obtained from Israeli National Blood Services following Tel Aviv University’s Institutional Review Board (IRB) approval. We imaged T-cells in three different stages: inactivated, activated 3 days old, and activated 7 days old. T-cells were isolated using the EasySep Direct Human Neutrophil Isolation Kit (StemCell Technologies, #19666, Vancouver, Canada) and the EasySep Direct Human lymphocytes CD4 + Isolation Kit (StemCell Technologies, #17952), following the manufacturer’s protocols. Initially, 0.5 mL of blood was transferred into 5-mL round-bottom polystyrene tubes, to which 50  μL of isolation cocktail and 50  μL of magnetic beads were added. The mixture was incubated at room temperature for 5 min, followed by the addition of 3.5 mL phosphate-buffered saline (PBS) containing 1 mM ethylenediaminetetraacetic acid (EDTA). After another 5 min of incubation, the tube was placed in a magnet (EasySep #18000) for 5 min, and the liquid was transferred to a new tube; 20  μL of isolation cocktails and 50  μL of magnetic beads were added to the tube, followed by incubating for 5 min, placing in the magnet for another 5 min, and transferring the liquid to a new tube for centrifugation at 1250 RPM (revolutions per minute). For the T-cell activation, the isolated cells were cultured in a six-well chamber with RPMI 1640 (01-100-1A, Sartorius, Göttingen, Germany) with 10% certified fetal bovine serum (Sartorius, # 04-001-1A). Activation was induced by adding 10  μL of Dynabeads human T-activator CD3/CD28 (#11161D, Thermos Fisher, Waltham, Massachusetts, United States) and incubating for 3 and 7 days in a 5% ${\mathrm{CO}}_{2}$-humidified incubator at 37°C. To confirm the presence of T-cells and activated T-cells, the cells were labeled with 5  μL of CD4/CD25 antibody cocktail, allophycocyanin, and fluorescein isothiocyanate (#22-0425-71, Affymetrix eBioscience, Santa Clara, California, United States). The cells were analyzed by flow cytometry using the CytoFlex LX system.

## 3 Optical System

**Fig. 1:**
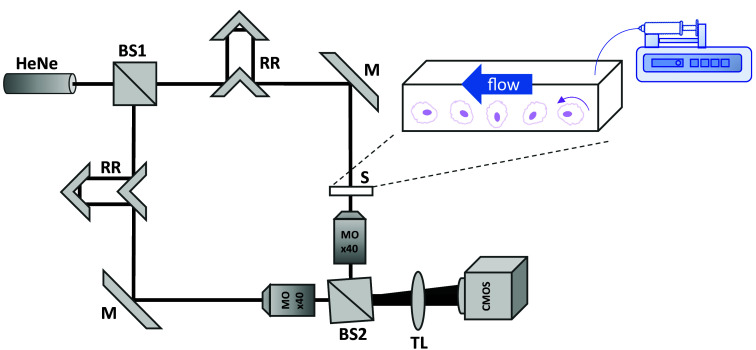
Label-free imaging flow cytometry setup based on a modified Mach–Zehnder microscope interferometer. BS1 and BS2, beam splitters; RR, retroreflectors; S, sample; MO, microscope objective; M, mirrors; CMOS, digital camera. A small tilt is induced at BS2 to create an off-axis image interferogram on the camera.

The cells were acquired by the modified Mach–Zehnder interferometer microscope illustrated in Fig. 1. The system was illuminated by a helium–neon laser source with a power of 17 mW, which was split at the first beam-splitter (BS1) into a sample beam and a reference beam. Both beams followed similar paths, directed by mirrors (M), and passed through a 40× microscope objective (MO, Leica, 440, 0.66 NA, Wetzlar, Germany). The optical path of the two beams was matched using retroreflectors (RR). The sample beam passed through the sample containing a 17  mm×1  mm W 0.1 mm deep channel (IBIDI, μSlide V0.1, Gräfelfing, Germany) with flowing cells. Both beams were recombined at the second beam-splitter (BS2) with a small off-axis angle between the sample and reference beams to produce an off-axis hologram. Both BS1 and BS2 had a split ratio of 50/50. The beams continued through a tube lens (TL, f=200  mm) and interfered with the image plane on a CMOS camera (IDS, 306CP-Rev.2) with a field of view of 284  μm×178  μm, sampling at 30  frames/s with an exposure time of 0.033 s. The flow within the channel with the rolling cells was generated by a low-pressure syringe pump (Cetoni, neMESYS 290N, Korbußen, Germany) set to a slow flow of $∼90\;\mu L\;h^{-1}$ on the bottom of the channel. The cells rolled freely along the bottom of the channel, eliminating focusing problems by capturing all the cells on the same focus plane, eliminating the need to use other mechanisms such as hydrodynamic focusing. Utilizing cell concentrations higher than $1000\text{ cells }\mu L^{-1}$ resulted in a throughput of ∼25  cells/s.

## 4 Dataset Preparation

**Fig. 2:**
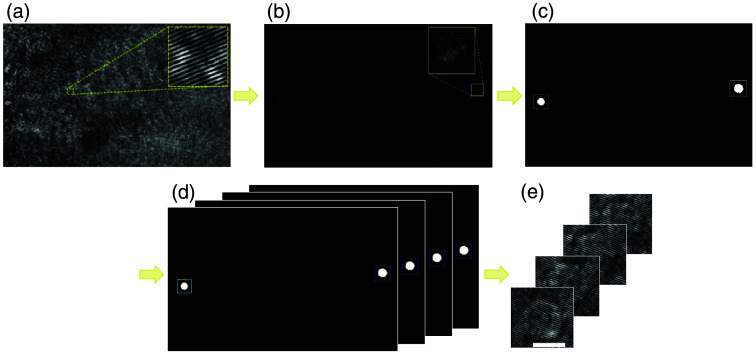
Process of cropping the cell holographic projections from the off-axis holographic video of flowing cells. (a) Entire off-axis hologram, where the yellow box shows a zoomed-in area. (b) After subtracting the background from the off-axis hologram, for detecting the cell location, where the yellow box shows a zoomed-in cell. (c) After binarization, dilation, hole filling, and segmentation, indicating the cell area. (d) Cell tracked over time. (e) Cropped holographic projections of cells, used as the inputs to the classifying neural network.

Up to 10 holographic projections were used per cell and classified directly by the deep neural network. The process of cropping the interferometric projections images of each cell from the video of the off-axis image hologram of flowing cells is described in Fig. 2. For every recorded off-axis hologram video, we first created a background image (frame without a cell present) by averaging all the frames in the video. The background image was subtracted from each frame containing the off-axis hologram to remove interference fringes, resulting in a clearer object image that allowed cell segmentation. The resulting image was binarized. We then applied shape dilation using a disk-shaped structural element with a diameter equivalent to the fringe frequency, followed by hole filling. Afterward, we used the “regionprops” function in MATLAB to compute the area, eccentricity, major and minor axis length, and circularity. We then segmented only the cells fully contained within the frame and satisfied the criteria of area (300 to 7100 pixels), axes ratio (>0.62), circularity (>0.26), eccentricity (<0.85), and cell size (38 to 120 pixels). The boundaries were manually chosen after examining the distribution of these parameters. The next step was creating projections set for every cell, which involved associating different frames with the same cell. As cells in the videos primarily moved along the flow axis, the same cell displayed slight movement along the perpendicular axis and more significant movement along the flow axis. For each cell, the software automatically assessed whether the distances between the centers of the cells along the perpendicular axis were sufficiently small (<21  pixels) and if the distances across the flow axis were sufficiently close (<480  pixels) among different frames. In addition, we checked if the cells had similar characteristics, such as area (<890  pixels), minor axis length (<14  pixels), and major axis length (<21  pixels). These numbers of pixels are set once per experiment. After that, we cropped and grouped all frames that met these criteria and applied zero-padded to ensure they were all the same size, 134×134  pixels, as the largest cell in the dataset.

**Fig. 3:**
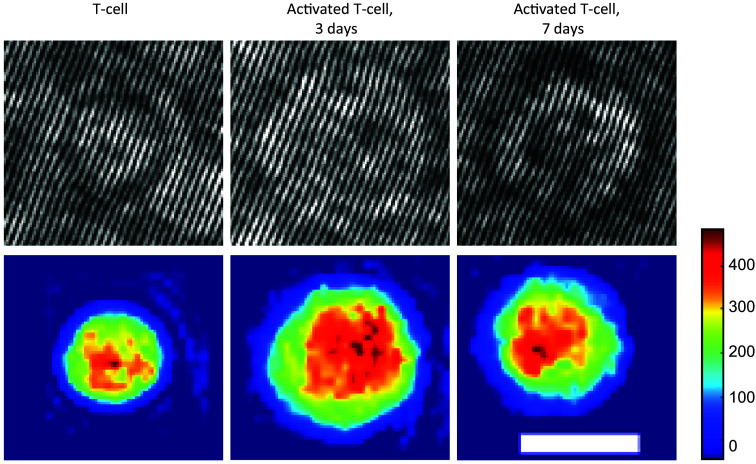
Example of the cell types acquired. First row, off-axis image holograms; second row, quantitative OPD images. The color bar represents the OPD values in nanometers. The white scale bar indicates 5  μm.

For the verification and comparison, we also reconstructed the OPD profiles of the cells. First, a two-dimensional (2D) Fourier transform was performed on each cell hologram image and cropped around one of the cross-correlation terms. Then, an inverse 2D Fourier transform was applied to the complex wavefront of the image and divided by a background complex wavefront matrix previously mentioned to remove aberrations and noise. This result is the complex wavefront of the cell. To create the OPD image, the phase of the resulting complex wavefront was unwrapped numerically,^32^ multiplied by the central wavelength of the light source and divided by 2π. Examples from the dataset are presented in Fig. 3.

## 5 Neural Network Architecture Training and Testing

**Fig. 4:**
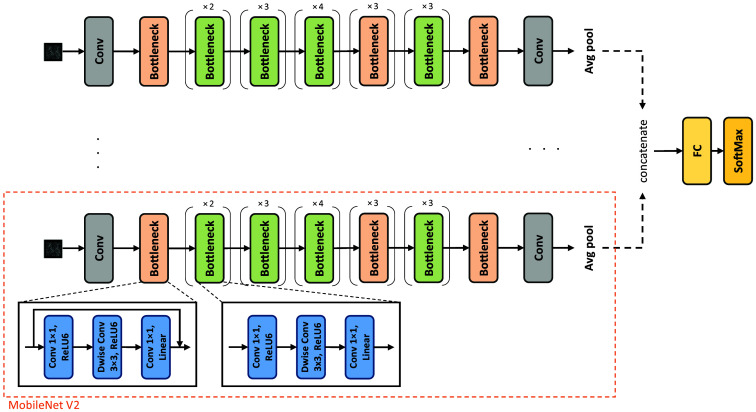
Network architecture involves passing each projection through the MobileNetV2 backbone (orange dash box). The results from each projection are concatenated and fed into a fully connected layer followed by SoftMax.

The CNN architecture used in our study is MobileNetV2, based on an inverted residual structure, where the input and output of the residual block are thin bottleneck layers, as shown in the orange dashed box in Fig. 4. This architecture mainly comprises two types of blocks, one is bottleneck block with a stride of 1, and the other with a stride of 2. In the case of a stride of 2, the block does not include a shortcut connection. Both blocks rely on depth-wise separable convolutions, which consist of two separate operations. The first, depth-wise convolution, applies spatial convolution independently over each channel of the input. This operation captures spatial information independently for each input channel. The second, point-wise convolution, applies a 1×1 convolution over all the individual feature maps created by the depth-wise convolution operation to combine them. This operation helps capture cross-channel correlations. These two operations are executed sequentially. Each block contains three layers, a 1×1 convolution with ReLU6, followed by depth-wise convolution with ReLU6, and finally another 1×1 convolution without any non-linearity reducing the risk of information loss due to nonlinear transformations. This structure enables the capture and representation of complex patterns in the input and leading to richer feature representations while reducing computational complexity. The incorporation of residual connections allows gradients to flow more easily during training.^33^

As the holographic projections contain different perspectives on the cell, we would like to process each one independently (late fusion). This approach allows for the characterization and differentiation of the distinctive features present in each projection. By doing so, we aim to create a comprehensive representation that integrates the various projections. 2D convolutions are not invariant to image rotation, and the filters are fixed in their spatial orientation and detect features at a specific location within the input image. Therefore, it may impair the results if we pass a single input containing all projections together through the network (early fusion). To achieve this, we employ a technique known as late fusion, which involves the aggregation of decisions from multiple classifiers, each of which is trained separately on its respective data or modality.^34^

Training a single MobileNet backbone with one input containing all projections together resulted in no significant changes in accuracy, despite increasing the number of projections. To process multiple projections simultaneously, we constructed a network with multiple heads, each based on the MobileNetV2 backbone, as shown in the orange dashed box in Fig. 4. The architecture passes each projection through a specific MobileNetV2 head; hence, each head processes one of the projections independently, allowing for each head to perceive a unique view of the cell. The outputs from the latent space of each head are concatenated and passed into a fully connected layer, followed by a SoftMax classification function, as illustrated in Fig. 4. After training 10 different models each with unique projection selection, we did not obtain performance differences depending on the selected projection.

## 6 Implementation Details

**Fig. 5:**
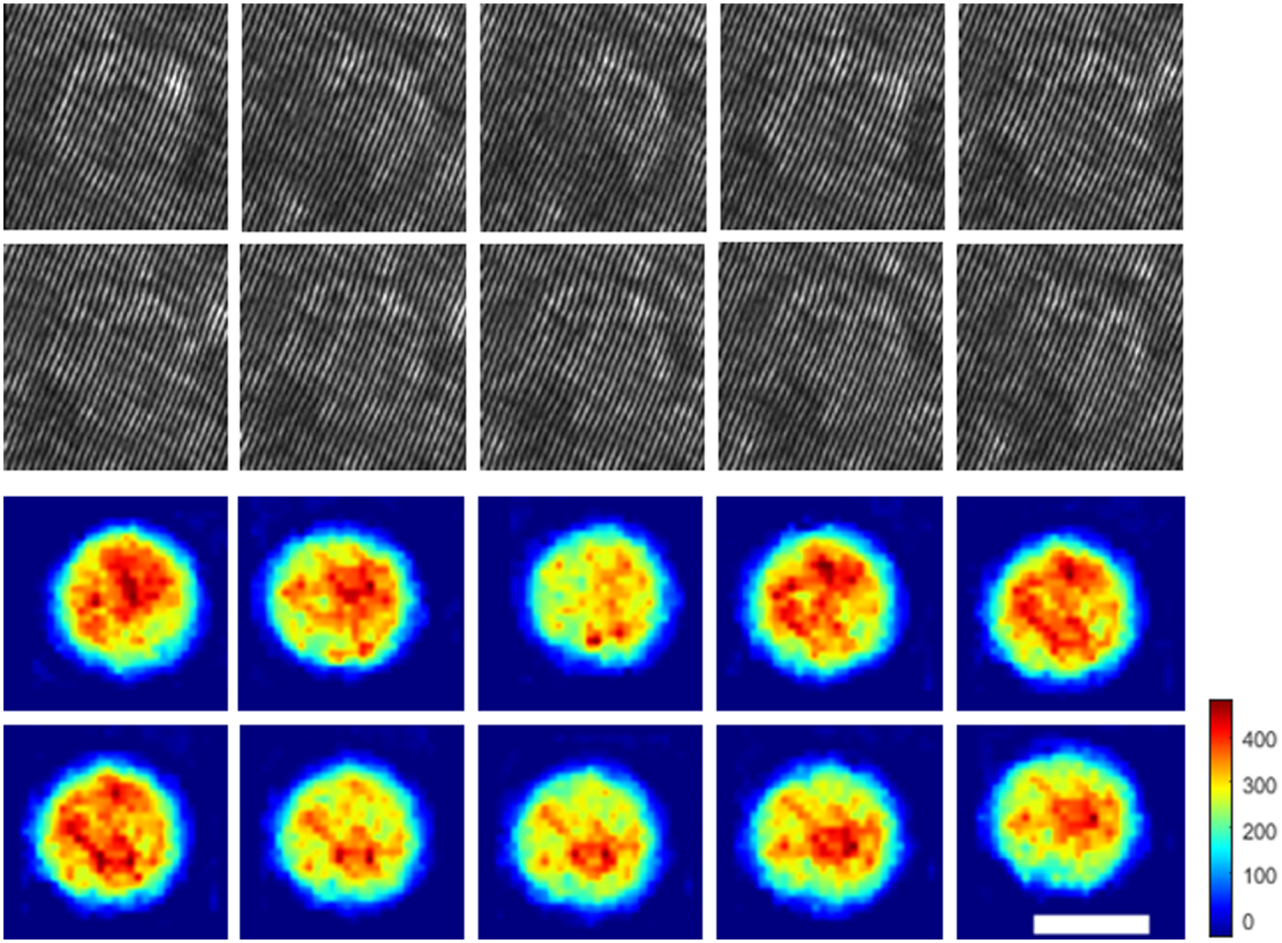
Example of 10 projections of the same activated T-cell, 7 days old. First two rows, off-axis hologram rolling set; last two rows, OPD rolling set. The color bar represents the OPD values in nanometers. The white scale bar indicates 5  μm.

The dataset consists of three types of T-cells, at different activation stages: 391 T-cells before activation, 387 activated T-cells, 3 days old, and 403 activated T-cells, 7 days old. Each cell is represented by 10 different projections, with Fig. 5 showing an example of the projections of an activated T-cell, 7 days old. The dataset was split into train set (80%), validation set (10%), and test set (10%) via fivefold cross-validation.

We first trained the network to be insensitive to changes in fringe frequencies and fringe directions by synthetically creating new off-axis holographic images with different off-axis angles and fringe direction angles and providing these augmentations to the network for training as described in the previous study.^30^ The training parameters included a batch size of 64, a constant learning rate of 0.0005, 10 epochs, and cross-entropy loss optimization using the Adam optimizer. Afterward, we employed this invariant fringe–spatial–frequency network to train a model directly on the raw off-axis holograms described above. The training parameters for this stage included a batch size of 64, a constant learning rate of 0.0001, 20 epochs, and cross-entropy loss optimization using the Adam optimizer.

For comparison and validation of the deep network, we trained MobileNetV2 on the OPD profiles as well. The training parameters included a batch size of 64, a constant learning rate of 0.000001, 30 epochs, and cross-entropy loss optimization using the Adam optimizer. Higher learning rates did not result in model convergence.

## 7 Results

**Fig. 6:**
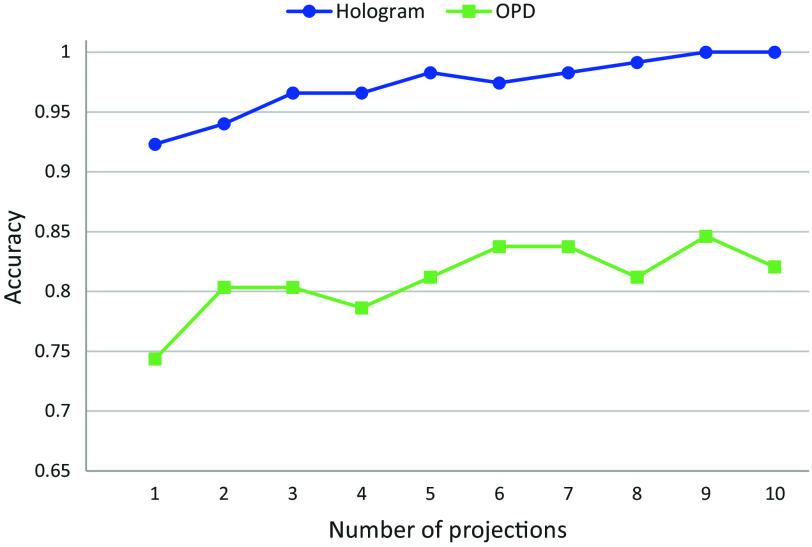
Test accuracies for classifying the three different activation stages of T-cells while increasing the number of projections for each cell. The blue line with circles is for the holographic projections; the light green line with squares is for OPD projections.

The results shown in Fig. 6 demonstrate a clear trend of increasing accuracy with the addition of more projections. Utilizing the raw holograms directly, we achieved an accuracy of 92.31% with one projection, and it increased to 100% with ten projections. In contrast, for the OPD profiles, we achieved an accuracy of 74.36% for one projection and 82.05% for 10 projections. Furthermore, all accuracy results for different numbers of projections were lower compared with the accuracy obtained with a single projection of the digital hologram.

**Fig. 7:**
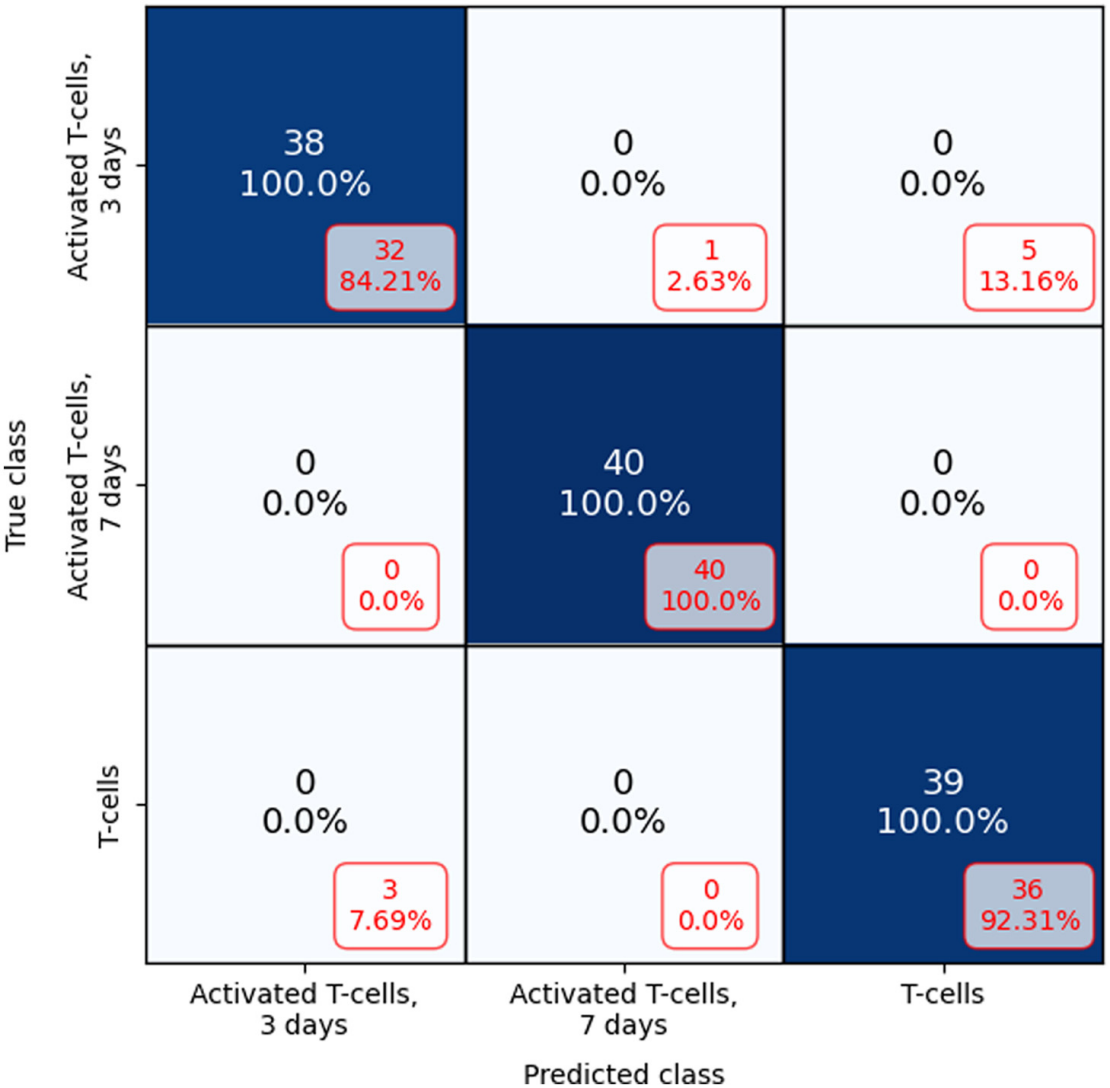
Confusion matrix for classification using 10 raw holographic projections compared with one raw holographic projection (in red, boxed) for three different activation stages of T-cells.

Figure 7 displays the confusion matrix that describes the classification results, comparing the use of 10 holographic projections to a single holographic projection. When employing a single holographic projection, the model erroneously classified 2.3% of activated T-cells, 3 days old as activated T-cells, 7 days old and 13.16% of them as inactivated T-cells. However, when increasing the number of projections to ten, the model successfully classified them correctly. For T-cells 7 days old, the model correctly classifies them whether using a single projection or multiple projections. For T-cells, the model misclassified 7.69% of them as activated for 3 days when using one projection but achieved the correct classification for 10 projections.

To demonstrate that the improvement in performance when directly processing the hologram is due to the inclusion of amplitude information, we trained a model using both the processed amplitude and OPD images through early fusion, achieving an accuracy of 91.45%. This result is similar to the accuracy obtained when processing directly the raw holograms.

**Fig. 8:**
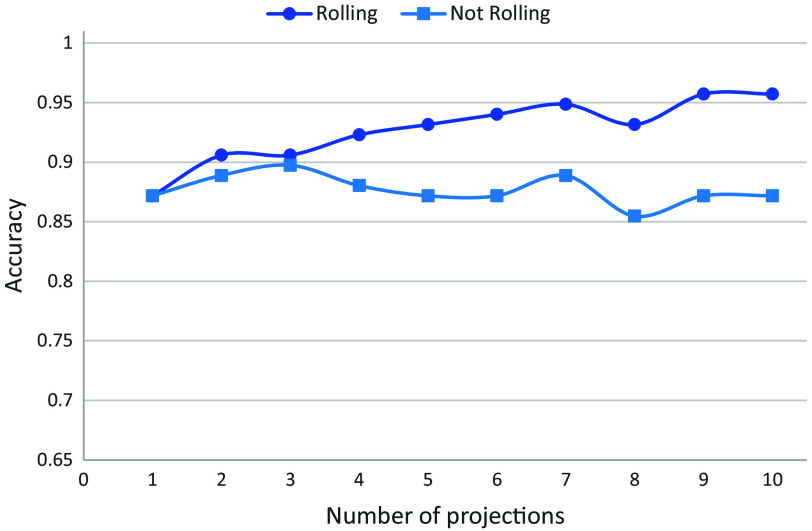
Test accuracies for classifying synthetic holograms of three different activation stages of T-cells while increasing the number of projections for each cell. The dark blue line with circles is for rolling cells (multiple cell projections); the light blue line with squares is for non-rolling cells (multiple cell exposures from the same perspective (not projections)).

We also compared analyzing exposures during a simple flow model of cells to analyzing cell projections during rolling. Figure 8 demonstrates the improvement of performance obtained when using multiple interferometric projections rather than using multiple cell exposures during flow. Specifically, on the rolling cell dataset, we achieved an accuracy of 87.18% with one projection, and it increased to 95.73% with 10 projections. For the non-rolling cell dataset, we achieved an accuracy of 87.18%, for both 1 and 10 projections, and this value was approximately maintained for any number of projections.

The inference time for the multiple MobileNet heads, performed with the Google Colab platform equipped with Tesla T4 GPU, is summarized in Table 1. These results indicate that with dedicated hardware and without the need for external storage for the images, it is possible to achieve even higher throughputs.

**Table 1:** Inference times for processing multiple projections.

No. of projections	1	2	3	4	5	6	7	8	9	10
Inference time (s)	0.00585	0.0138	0.0215	0.0264	0.0322	0.0316	0.0377	0.0406	0.0465	0.0591

**Fig. 9:**
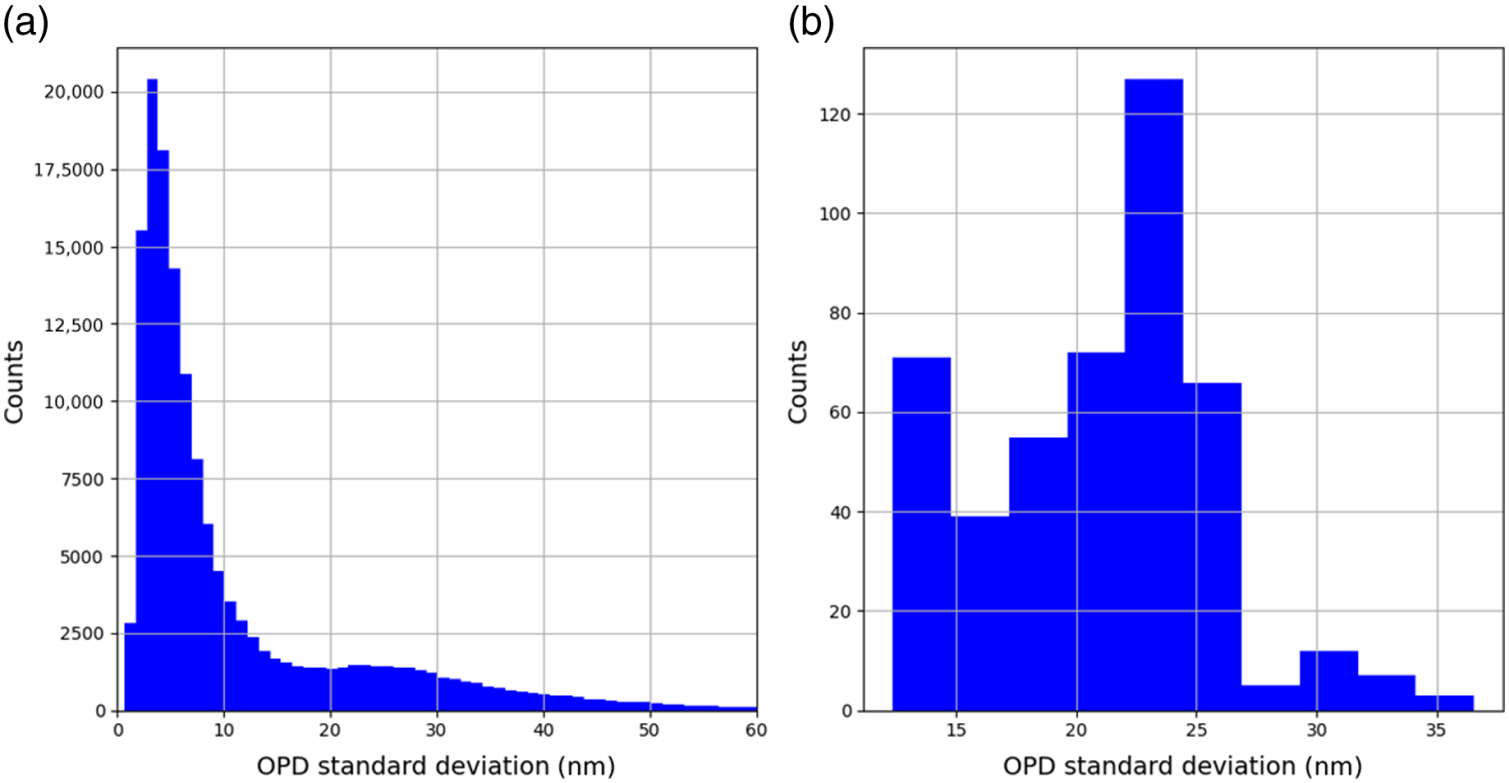
(a) OPD temporal stability. (b) OPD spatial stability.

Figure 9(a) presents the standard deviation distribution of the OPD maps across time, with a mean temporal stability of 12.22 nm, and Fig. 9(b) presents the standard deviation distribution of OPD maps across space, with a mean spatial stability of 21.05 nm.

## 8 Discussion and Conclusion

In this work, we presented a new approach for classifying cells based on multiple digital hologram projections. We created a network with multiple classifiers to process each projection independently and integrated them together in the end. Our findings indicate that increasing the number of projections enhances the ability of the model to distinguish among different types of T-cells. The additional information resulting from these added projections increases the robustness of the cells and allows more precise characterization of the various activation stages of the T-cells.

The utilization of digital holograms instead of the OPD profiles highlights the effectiveness of combining amplitude and phase information for comprehensive characterization and yields better results even with just a single viewing angle of the cell. The high accuracy achieved through multiple individual projections without the need for tomographic processing allows for significant savings in computational resources and achieves faster classification.

The paper presents a low-throughout proof-of-principle imaging flow cytometry experiment, rather than demonstrating clinical imaging flow cytometry in thousands of frames per second, which would require more efficient cameras and microfluidic systems. However, its adaptation to these systems is straightforward, because all the processing is done on the raw holograms and using the random rotation of the cells during flow. Given the changes in the camera and the microfluidic system, which will affect the pixel size, the number of pixels in the image, and the speed of cell flow, it will be necessary to adjust the thresholds defined in the algorithms for identifying cells and finding their projections across different frames. This adjustment is done manually once per experiment and is relatively straightforward through examination of the cells. The rest of the process is automatic.

In conclusion, we demonstrated that employing several different perspective views of the cell through holographic imaging leads to increased accuracy in cell classification tasks, especially for cells that closely resemble each other such as T-cells in different activation stages. The proposed method enables highly informative and direct cell classification using deep learning without full tomographic reconstruction, therefore reducing time and computational complexity. In the future, following offline training of the deep neural network, implementation of the classifying deep neural network in a dedicated hardware based on a single-channel imaging of label-free flowing cells, without the need for an external computer or storing the images of the flowing cells in the computer, is expected to allow integration of real-time, label-free cell classification into the imaging cell cytometry system, obtaining much higher throughputs possible today.

## Data Availability

The data and codes underlying the results presented in this paper are not publicly available at this time but may be obtained from the authors upon reasonable request.
